# Diversity of ‘Cabernet Sauvignon’ Grape Epidermis and Environmental Bacteria in Wineries from Different Sub-Regions of the Eastern Foothills of Helan Mountain, Ningxia

**DOI:** 10.3390/foods13020252

**Published:** 2024-01-12

**Authors:** Hui Yang, Zheng Wang, Zhong Zhang, Chao Shu, Jiaqi Zhu, Ying Li, Junxiang Zhang

**Affiliations:** 1 School of Life Sciences, Ningxia University, Yinchuan 750021, China; yanghui15595195396@163.com (H.Y.); zhangzhongnxu@126.com (Z.Z.); 2 Institute of Medical Sciences, Ningxia Medical University, Yinchuan 750004, China; 3 School of Wine & Horticulture, Ningxia University, Yinchuan 750021, China; w17772159889@163.com (Z.W.); shuchao1016@163.com (C.S.); zhujiaqi001011@163.com (J.Z.); binggongchangli@yeah.net (Y.L.); 4 Engineering Research Center of Grape and Wine, Ministry of Education, Yinchuan 750021, China

**Keywords:** Eastern Foothills of Helan Mountain, Cabernet Sauvignon, epidermis of wine grapes, winery, bacteria

## Abstract

Understanding the composition of the bacterial community on the epidermis of wine grapes and in winery environments, as well as the response of grape epidermal bacteria to climatic factors, plays a significant role in ensuring grape health and promoting grape conversion into wine. This study utilized high-throughput sequencing to explore the composition of the bacterial community on the wine grape epidermis and representative wineries of three sub-regions of the Eastern Foothills of Helan Mountain, Ningxia. The results showed that the bacterial diversity and richness in the Yongning (YN) sub-region were the highest, with Qingtongxia (QTX) having the lowest levels of grape epidermal bacteria. The bacterial diversity and richness were the highest in Yinchuan (YC) and the lowest in YN in the winery environment (*p* < 0.05). The composition of dominant bacteria on the grape epidermis and in winery environments of the three sub-regions was not different at the phylum and genus level, but the levels of these dominant bacteria were different among the sub-regions. There was a correlation between grape epidermal bacteria and climatic factors. Approximately 93% of the bacterial genera on the grape epidermal genera in the three sub-regions are present in the winery environment and contain all the dominant bacterial genera on the epidermis.

## 1. Introduction

Due to the interaction between grapes and different growing conditions, including climate, soil, topography, agricultural management and winemaking processes, wines made from the same grape variety grown in different regions will exhibit different characteristics [1]. These interactions affect the expression of wine terroir [2,3,4]. Thus, terroir is an important aspect of consumer acceptance, recognition and economic value in wine production. However, the microbiome, composed of local fungi and bacteria, may represent another factor affecting the characteristics of regional wines [5].

Studies have shown that the diversity levels of bacteria found in vineyards and wineries are higher than those of fungi [6]. However, the role of many bacteria present on the surface of grapes in wine production, such as *Enterobacterium*, *Enterococcus*, *Bacillus*, *Burkholderia*, *SerratiaBizio*, *Staphylococcus*, etc. [7], is mostly ignored [8]; although they are unable to survive the extreme conditions of wine fermentation, their metabolic activity on grape surfaces may have long-term consequences for wine products [9,10]. High-throughput sequencing technology (HTS) enables the detection and quantification of microorganisms present on the grape epidermis and their subsequent transformation in the winery [11,12,13]. HTS can help us understand the much larger microbial diversity than traditional microbial culture methods, and it is possible to collect large amounts of high-precision and low-cost data to study microorganisms that cannot be cultured or have not yet been identified [14]. At present, the main bacterial phyla detected on the surface of grapes include Proteobacteria, Firmicutes, Actinobacteria, Bacteroidetes, etc. [15]. The dominant bacteria genera include *Acinetobacter*, *Methylobacterium*, *Massilia*, *Pantoea*, *Pseudomonas*, *Bacillus*, *Brevundimonas* and *Sphingomonas* [3,11,16,17,18,19,20]. However, the microbial diversity and stability of grape epidermis, as a natural habitat for microorganisms, are closely related to many factors, such as the altitude, latitude and longitude of the vineyard [21], and climatic conditions, such as rain, temperature, humidity, etc. [22]. Moreover, the use of agrochemicals, such as pesticides in vineyard management, can affect the composition of the microbial community on the grape surface [23].

After the grapes are harvested, the grape clusters arrive at the winery, and the bacterial communities originating from the grape skin from the first microbial input determine which species affect the wine’s flavor characteristics [24]. When the grape juice comes into contact with different winemaking equipment, bacteria that come into contact with these surfaces will enter the grape juice and begin to multiply when environmental conditions permit [25]. Currently, a number of bacterial genera belonging to at least 25 phyla have been detected in the brewery ecosystem; prominent examples include Proteobacteria, Firmicutes, Actinobacteria and Bacteroides [11,12,17,26,27,28].

Recent studies have shown that bacteria in the grape epidermis can affect the quality of wine when the fermentation process begins in the wine environment [29,30]. The Eastern Foothills of Helan Mountain in Ningxia represent a protected area in China for the production of wine products and are home to a large number of microbial resources; however, the diversity of bacterial communities in the skin and representative winery environments of Cabernet Sauvignon grapes in different sub-regions of the Eastern Foothills of Helan Mountain has not been analyzed or studied. Therefore, this study characterized the bacterial diversity of the grape epidermis and the winery environment and explored the relationship between the winery environment and the bacterial community found in the grape epidermis in different sub-regions of the area.

## 2. Materials and Methods

### 2.1. Location Description and Sampling

Cabernet Sauvignon samples were harvested from vineyards in the Eastern Foothills of Helan Mountain, Ningxia, between 26 September and 2 October 2022 (Figure S1). Yinchuan YUANSHI Winery (YC, 106°05′ N, 38°56′ E, 8-year-old vineyard), Yongning Lilan Winery (YN, 105°97′ N, 38°28′ E, 12-year-old vineyard) and Qingtongxia West Pigeon Winery (QTX, 105°88′ N, 38°08′ E, 8-year-old vineyard). From 1 April to 30 October 2022, the sunshine hours (h), raining (mm), average temperature (°C), wind speed (m/s) and relative moisture (%) were surveyed daily. All meteorological data were provided by Ningxia Meteorological Bureau (Table S1).

### 2.2. Grape Epidermal Sampling

The sample size was 1000 berries or equivalent bunches. The upper, middle and lower parts of the grape bunches were wiped with a sterilized cotton swab sampling stick dipped in sterile saline [29], with care taken to ensure that the side exposed to sunlight and the shaded side of each bunch were sampled. Each sampling point was set to repeat the test three times, with samples for each test being collected from five sampling plots in the vineyard. Then, the cotton swab head was cut off with sterilized scissors and put into a sterile tin foil bag for mixing. Then, the foil bag was blasted in liquid nitrogen and brought back to the lab for storage at −80 °C [20].

### 2.3. Sampling of Winery Environment

During the harvest period from September to October, winery environment samples were collected from the representative wineries in the three main sub-regions of the Eastern Foothills of Helan Mountain. The fermentation tank mouth, sink stem removal crusher, air bag press, conveyor belt, and ground were wiped with a sterilized cotton swab sampling rod for microbial sampling [29], and each sampling point was set to repeat the test three times. Samples were put into a sterile tin foil bag and the sample was mixed and put into a tin foil bag in liquid nitrogen for quick freezing before being brought back to the laboratory for storage at −80 °C

### 2.4. DNA Extraction and PCR Amplification

After thawing, each sample was placed in a 50 mL sterilized centrifuge tube, and 20 mL of sterile normal saline was added [29], and then each tube was placed in an ultrasonic cleaner for 6 min (20 °C). Samples were then taken out and placed in a constant-temperature shaking box for 20 min (200 rpm, 20 °C) before being placed in an ultrasonic cleaner for 3 min. The microorganisms in the samples were then eluted into normal saline; finally, the samples were placed in a centrifuge at 12,000 rpm/min, and the bottom precipitate was collected after centrifugation for 15 min for DNA extraction [31]. Total DNA was extracted using an E.Z.N.A. Soil DNA Extraction Kit (Omega Bio-tek, Norcross, GA, USA), and the DNA quality, concentration, and purity were checked using 1% agarose gel electrophoresis and NanoDrop2000. The extracted DNA was used as a PCR amplification template, and PCR primers were used for PCR amplification of the 16S rRNA V3–V4 variable region using 338F (5′-ACTCCTACGGGAGGCAGCAG-3′) and 806R (5′-GGACTACHVGGGTWTCTAAT-3′) [5]. Amplification was performed in 5× of TransStart FastPfu buffer (4 μL), 2.5 mM dNTPs (2 μL), TransStart FastPfu DNA polymerase (0.4 μL), 5 μM upstream and downstream primers (0.8 μL each), 10 ng template DNA, and a PCR system supplemented to 20 μL using ddH_2_O, with 3 replicates per sample [32]. The amplification procedures were 95 °C for 3 min and 27 cycles of 95 °C for 30 s, 55 °C for 30 s, 72 °C for 45 s, and a final extension at 72 °C for 10 min.

### 2.5. Illumina MiSeq Sequencing

Gels with 2% agarose were used for PCR product recovery, AxyPrep DNA Gel Extraction Kit was used for DNA purification, and 2% agarose gel electrophoresis and Quantus™ fluorometer (Promega, Madison, WI, USA) were used for DNA detection and quantification. The NEXTFLEX Rapid DNA-Seq Kit was used for library preparation, and Illumina’s Miseq PE300/NovaSeq PE250 platform (Majorbio Bio-pharm Technology Co., Ltd., Shanghai, China) was used for sequencing. The raw data are uploaded to the NCBI (accession number: SRP462062).

### 2.6. Processing of Sequencing Data

FASTP [33] was used for raw sequencing QC and FLASH [34] was used for splicing. According to the similarity [35,36], the sequences were OTU clustered and chimeras were eliminated using UPARSE software (http://drive5.com/uparse/ accessed on 22 February 2023, version 7.1). The RDP classifier [37] was used for classification annotation to be consistent with the Silva 16S rRNA database (v138) and the alignment threshold was set to 70%. OriginPro 2021 software (OriginLab Corporation, Northampton, MA, USA) were used for statistical analyses in this paper. Samples were analyzed using the Kruskal–Wallis H test in the R4.2.2 project Vegan software package.

## 3. Results

### 3.1. Evaluation of Bacterial Community Diversity on the Grape Epidermis and in Winery Environments

The glucose contents of Cabernet Sauvignon grapes harvested from QTX, YN, and YC were 226.77, 227.74, and 229.67 g/L, respectively; the titratable acidity levels were 5.9, 4.8, and 5.6 g/L, respectively (GB/T 15038-2006200-6) [38]; and the pH values were 3.40, 3.55, and 3.65, respectively. Samples of grape skins were obtained at the ripening stage of Cabernet Sauvignon grapes from the three sub-regions.

**Figure 1:**
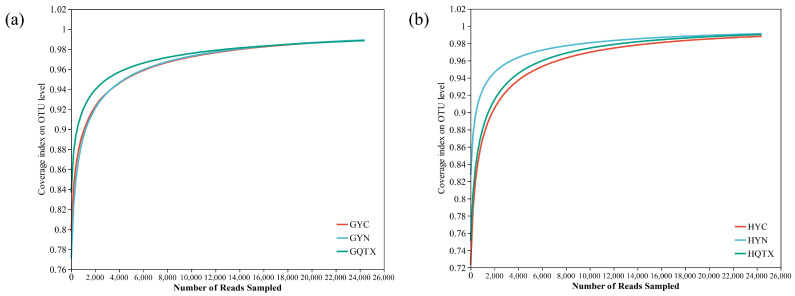
Dilution curves. (**a**) Dilution curves of grape epidermal bacterial communities in three sub-regions; (**b**) dilution curves of bacterial communities in the winery environment in the three sub-producing areas. *X*-axis, sequencing reads for each sub-appellation; *Y*-axis, the percentage of bacteria found in each subsample out of the total number of bacteria predicted.

The bacterial diversity of Cabernet Sauvignon grape epidermis samples and winery environment samples from three sub-regions of the Eastern Foothills of Helan Mountain were determined. A total of 2,772,527 optimized sequences were obtained via high-throughput sequencing. A total of 116,672 high-traits bacterial V3–V4 Illumina series were received from nine grape epidermis samples, with an average length of 420 bp. A total of 35,266, 36,048, and 45,358 high-quality labels were detected in the YC, YN, and QTX regions, and 887, 901, and 752 bacterial OTUs were obtained, respectively. The sparsity curves of all samples reached a plateau with bacterial coverage of more than 99% (Figure 1a), showing that the serial deepness of the specimen represent the true picture of the bacterial community on the grape epidermis and met the analytical requirements. A total of 2,655,858 high-quality bacterial V3–V4 Illumina series were acquired from 54 samples taken from the studied winery environments, with an average length of 416 bp. The number of high-quality labels of bacterial communities detected in the YC, YN, and QTX production areas was 878,081, 997,897, and 779,880, respectively, and the number of bacterial OTUs obtained was 18,884, 11,665, and 16,363, respectively. The bacterial coverage of all samples was more than 99%, and the sparsity curves tended to be flat (Figure 1b), showing that the series depth of the specimens could reflect the true situation of the bacterial community in the winery environment and met the requirements of subsequent analysis.

The Shannon Index reflects species diversity, and the Chao 1 index and ACE index reflect species richness. There was no significant difference in the diversity and richness of bacterial communities in the three sub-regions, with the highest diversity and richness of bacterial communities in the YN sub-region (Shannon, Chao 1 and ACE indices of 4.15, 1121 and 1131, respectively) and the lowest in the QTX sub-region (Shannon, Chao 1 and ACE indices of 3.73, 1016 and 1066, respectively) (Table S2). However, comparing the diversity and richness of bacterial communities in the winery environment of the three sub-producing areas, it was found that the diversity and richness of bacterial communities in the YC sub-producing area were the highest (Shannon, Chao 1 and ACE indices were 4.41, 1299 and 1340, respectively), and the diversity of bacterial communities in this sub-producing area was significantly higher than that in the YN producing area (*p* < 0.05). The YN sub-region was the lowest (Shannon, Chao 1 and ACE indices were 3.43, 860 and 904, respectively) (Table S3). Spearman correlation analysis was performed on the Shannon, Chao 1, and ACE indices of grape skin and winery environment, and the results showed that the Shannon and Chao 1 indices for the grape epidermis were positively correlated with the fermentation tank mouth (*p* < 0.05). The ACE index was not obvious (Figure S2).

### 3.2. Analyze of Bacterial Community Consisting of Grape Epidermis in Various Sub-Producing areas

**Figure 2:**
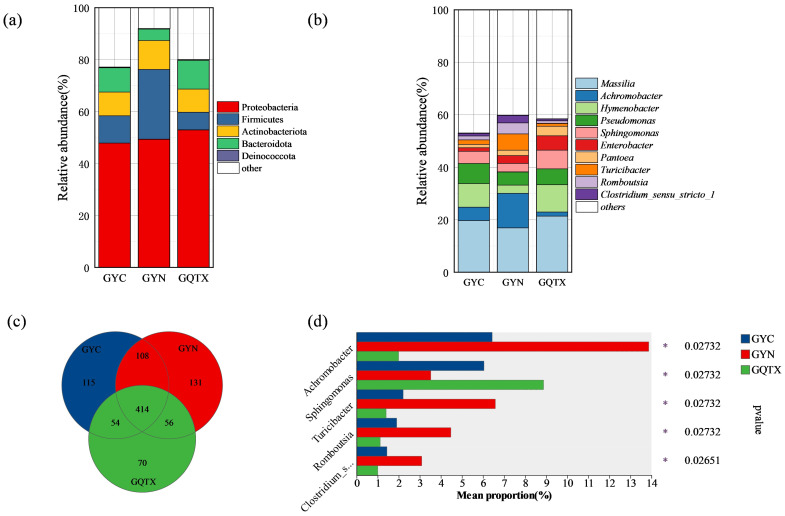
Bacterial diversity of grape epidermis in different sub-regions. (**a**) The phylum Bacteria; (**b**) the 10 most abundant bacteria with a relative abundance greater than 1% at the bacterial genus level; (**c**) Venn diagram showing bacterial genus levels; (**d**) bacterial genera that differed significantly between the three regions. * represents *p* < 0.05.

At the level of phylum, Proteobacteria is the advantage phylum among all samples, with an average richness of 50.04%. Firmicutes followed, accounting for 14.73% on average, with the highest abundance in YN (26.84%) (Figure 2a). At the genus level, 414 bacterial genera were shared by the three sub-regions, among which the YN sub-region had the largest number of bacterial genera (709) and the largest number of platoon genera (131) (Figure 2c).

Figure 2b shows the top 10 bacteria, representing three sub-regions at the genus level with a relative abundance of more than 1% in the grape epidermis. These include the following: *Massilia*, *Achromobacter*, *Hymenobacter*, *Pseudomonas*, *Sphingomonas*, *Enterobacter*, *Pantoea*, *Turicibacter*, *Romboutsia*, and *Clostridium Sensu_stricto_1*. *Massilia* was the dominant genus in all samples, followed by *Achromobacter*, *Achromobacter*, *Hymenobacter*, *Pseudomonas*, and *Sphingomonas*. The results showed that the dominant bacterial communities in the three sub-regions were similar in composition, but there were differences in relative abundance. The results of Figure 2d show that *Achromobacter*, *Sphingomonas*, *Turicibacter*, *Romboutsia*, and *Clostridium_sensu_stricto_1* had significant differences in YC, QTX, and YN (*p* < 0.05); *Achromobacter*, *Turicibacter*, *Romboutsia*, and *Clostridium_sensu_stricto_1* had the largest abundance in the YN sub-region; and *Sphingomonas* had the largest abundance in the QTX sub-region.

### 3.3. Correlation between Vineyard Climate and Bacterial Communities on the Grape Epidermis

**Figure 3:**
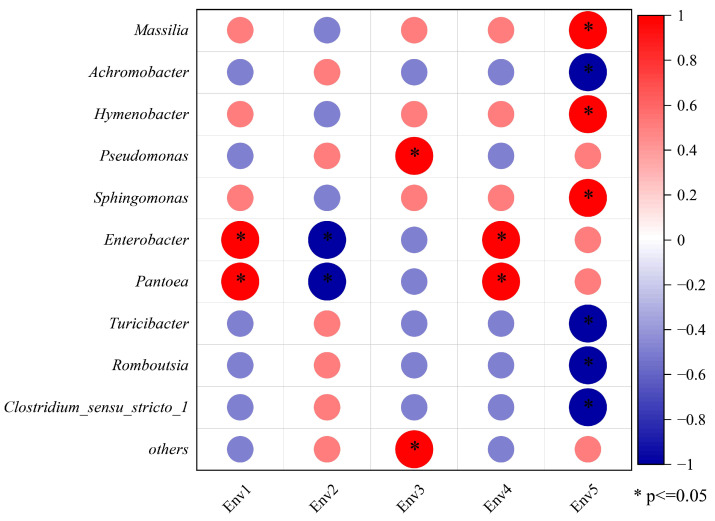
Correlation analysis of the top 10 bacterial genera with relative abundance of grape skin and meteorological parameters. A red circle with an asterisk indicates a positive correlation (Spearman’s rank test, *p* < 0.05) and a blue circle with an asterisk indicates a negative correlation (Spearman’s rank test, *p* < 0.05). Env1 represents the average temperature; Env2 represents the mean relative humidity; Env3 represents cumulative rainfall; Env4 represents the average wind speed; Env5 stands for the average number of hours of sunshine.

The microflora of the grape epidermis is tightly connected to various meteorological parameters. The relationship between grape epidermis microorganisms and meteorological parameters was studied using correlation analysis, and the results are shown in Figure 3. A correlation was found between grape epidermis bacteria and meteorological parameters (Spearman’s correlation coefficient *p* < 0.05). For example, the average temperature and average wind speed of vineyards were significantly positively correlated with *Enterobacter* and *pantoea*; the average humidity was negatively correlated with *Enterobacter* and *pantoea*; the cumulative rainfall was significantly positively correlated with *pseudomonas*; the average sunshine hours were significantly positively correlated with *Massilia*, *Hymenobacter*, and *Sphingomonas*; and the average sunshine hours were significantly positively correlated with *Massilia*, *Hymenobacter*, and *Sphingomonas* and significantly negatively correlated with *Achromobacter*, *Turicibacter*, *Romboutsia*, and *Clostridium_sensu_stricto_1*.

### 3.4. Environmental Bacterial Diversity of Wineries in Different Sub-Regions

**Figure 4:**
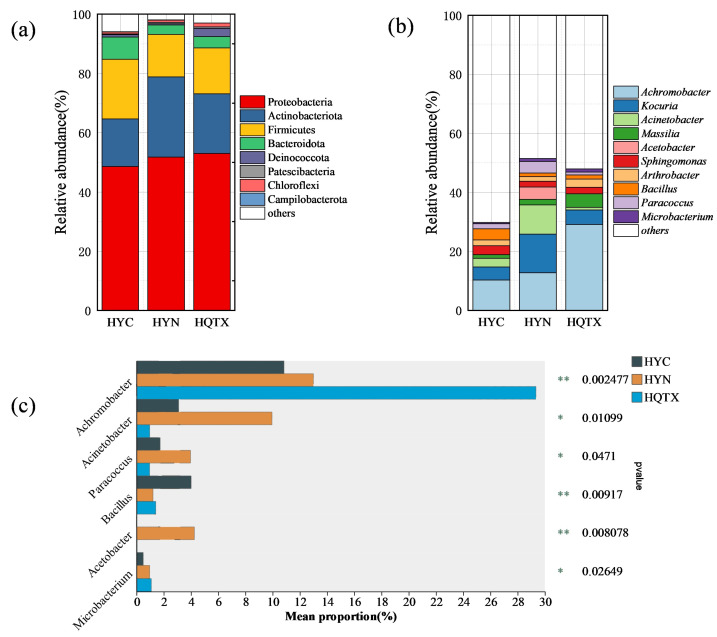
Relative abundance of bacterial communities in the winery environment at the phylum and genus levels. (**a**) The phylum bacteria; (**b**) the 10 most abundant bacteria with a relative abundance greater than 1% at the bacterial genus level; (**c**) bacterial genera that differ significantly between the three wineries. ** represents *p* < 0.001, * represents *p* < 0.05.

Proteobacteria was the leading phylum in all samples, with a mean abundance of 51.14%, with the maximum abundance in the QTX subregion (52.98%). Acinobacteriota (21.04%), Firmicutes (16.68%), and Bacteroidota (4.84%) had the highest abundance in the YN sub-producing area (26.84%) (Figure 4a). Figure 4b shows the 10 most abundant bacterial genera with a relative abundance greater than 1% in the winery environment in the three sub-regions at the genus level, which include the following: *Achromobacter*, *Kocuria*, *Acinetobacter*, *Massilia*, *Acetobacter*, *Sphingomonas*, *Arthrobacter*, *Bacillus*, *Paracoccus*, and *Microbacterium*. The results showed that the dominant bacterial communities in the winery environment of the three sub-regions were similar in composition, but there were differences in relative abundance. The results of the Kruskal–Wallis H test were used to analyze the significant differences between the 10 greatest richness species with a relative abundance larger than 1% at the bacterial genus level in the samples of the three sub-regions, and the results are shown in Figure 4c. *Achromobacter*, *Acinetobacter*, *Paracoccus*, *Bacillus*, *Acetobacter*, and *Microbacterium* demonstrated significant differences in YC, QTX, and YN. *Achromobacter* and *Microbacterium* had the highest relative abundances in the QTX sub-region. *Acinetobacter*, *Paracoccus*, and *Acetobacter* had the highest relative abundances in the YN sub-region, and *Bacillus* had the highest relative abundance in the YC sub-region.

**Figure 5:**
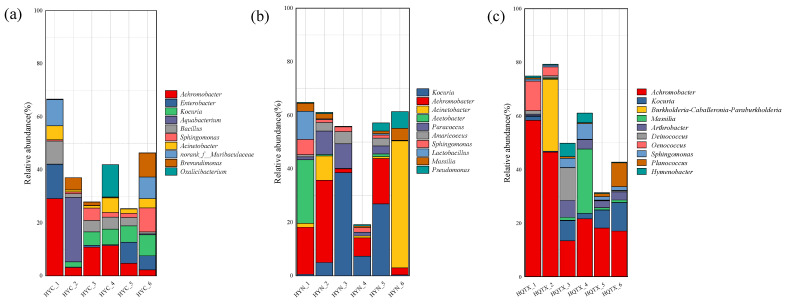
Composition of bacterial communities in the distillery environment in different sub-regions. (**a**) Yinchuan YUANSHI Winery; (**b**) Yongning Lilan Winery; (**c**) Qingtongxia West Pigeon Winery. (1) Representative sampling point for the mouth of the fermentation tank; (2) representative sampling point for the sink; (3) representative sampling point for the destemming crusher; (4) representative sampling point for the airbag press; (5) representative sampling point for the conveyor belt; (6) representative sampling point for the ground.

Figure 5 shows the bacterial composition and distribution of the environment of representative wineries in the three sub-producing areas of Helan Mountain. The 10 most abundant bacterial genera with a relative abundance greater than 1% in the bacterial composition of each winery are listed separately, and the results showed that the genus with the highest abundance in YC and QTX was *Achromobacter*, and the genus with the highest abundance in YN was *Kocuria*. *Achromobacter* was clearly enriched at the mouth of the fermenter in the three wineries, including *Oenococcus* at the mouth of the QTX production area (Figure 5c), *Acetobacter* at the mouth of the YN production area (Figure 5a), and *Lactobacillus* and *Sphingomonas* at the mouth of the YN distillery (Figure 5b). The genera enriched in destemming crushers, balloon presses, and conveyor belts were *Massilia*, *Sphingomonas*, *Acinetobacter*, and *Hymenobacter*. *Acinetobacter* enrichment was found in the fermenter mouth, air bag press, sink, conveyor belt, and floor in the YC and YN production areas, and *Enterobacter* was a dominant bacterial genus unique to the brewery environment in the YC production area, enriched in the fermentation tank mouth, conveyor belt, and ground.

**Figure 6:**
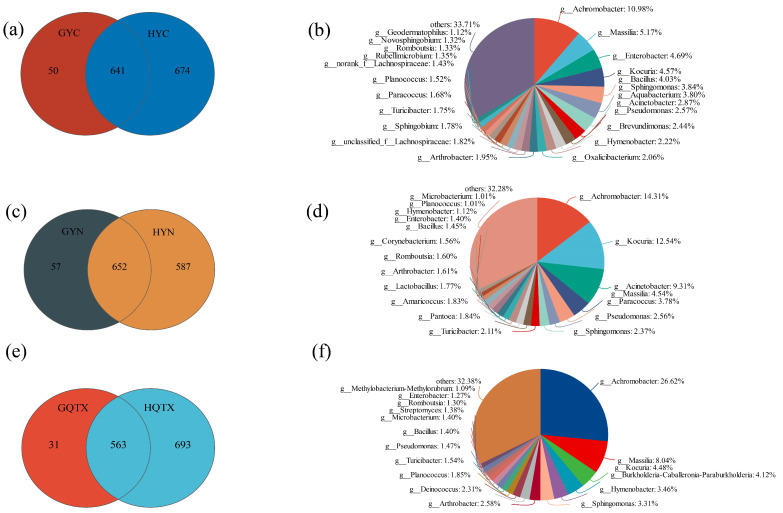
Venn diagram and pie chart at the level of bacterial genus in grape skin and winery environmental samples (**a**) Venn diagram of bacterial levels in grape skins and winery environmental samples in the YC sub-region; (**b**) In the YC sub-region, there were bacterial genera with a relative abundance of more than 1% in grape skins and winery environmental samples; (**c**) Venn diagram of bacterial levels in grape skins and winery environmental samples in the YN sub-region; (**d**) In the YN sub-region, there were bacterial genera with a relative abundance of more than 1% in grape skins and winery environmental samples; (**e**) Venn diagram of bacterial levels in grape skins and winery environmental samples in the QTX sub-region; (**f**) In the QTX sub-region, there were bacterial genera with a relative abundance of more than 1% in grape skins and winery environmental samples.

Figure 6 presents results of analysis of the relationship between the bacterial community of the grape epidermis and the bacterial community of the winery environment in different sub-regions; it was found that 92.76% (641) of the bacterial genera in the grape epidermis bacterial community in the YC production area also existed in the winery environment, and only 7.23% (50) of the genera were unique to the grape epidermis (Figure 6a); additionally, 91.96% (652) of the bacterial genera in the grape epidermis bacterial community in the YN production area also existed in the winery environment. Only 8.04% (57) of the genera were specific to the grape skin (Figure 6c), 94.78% (563) of the bacterial community of the grape skin in the QTX area also existed in the winery environment, only 0.51% (31) of the genera were specific to the grape epidermal (Figure 6e), 23 bacterial genera were more than 1% of the common bacterial genera on the grape epidermis and in the winery environment in the YC sub-region (Figure 6b), and the relative abundance of the common bacterial genera on the grape epidermis and in the winery environment in the YN sub-region was greater than 1%. A total of 19 bacterial genera (Figure 6d) and 17 bacterial genera were found to have a relative abundance greater than 1% of the common bacterial genera on the grape epidermis and in the winery environment in the QTX sub-region (Figure 6f), indicating that most of the bacteria on the grape epidermis will be brought to the winery environment and remain in the winery environment; this, of course, includes all dominant bacterial genera. Spearman correlation analysis was carried out on the bacterial communities of the grape epidermis (bacteria ranked in the top 10 for relative abundance) and the bacterial communities in the winery environment (bacteria ranked in the top 10 for relative abundance). The results showed that there was no significant correlation between the grape epidermis bacteria and the bacterial communities in the winery environment (Figure S3).

## 4. Discussion

Microorganisms play a vital part in both the quality and yield of grapes, as well as the quality of wine. Since 99% of microorganisms are non-culturable, traditional microbial culture techniques limit the understanding of microbial diversity [39]. However, molecular biology techniques, particularly HTS, have created opportunities to understand non-culturable, low-content, and unknown microbial communities [14,18,39,40]. In this study, HTS was used to amplify and sequence the V3–V4 regions of the bacterial 16 S rRNA gene in the grape epidermis and winery environment in three sub-regions of Helan Eastern Foothills in Ningxia. The bacterial diversity and richness of the grape epidermis were the highest in YN and the lowest in QTX. The diversity and richness of the bacterial community composition of the epidermis of Cabernet Sauvignon grapes may be due to the geographical location of the appellation, and it has been previously found that the microbial community of the epidermis of wine grapes is affected by geographical factors [36]. Feifei Gaoa et al. found that there were obvious differences in the bacterial composition of grapes on the epidermis of grapes in six wine-producing areas in Xinjiang, and the reason for this difference was related to the geographical environment [21]. Reguant and Bordons et al. used high-throughput sequencing to explore the bacterial diversity on the skin of grapes of two grape varieties (Grenache and Grenache), and the results showed that differences in bacterial communities were affected by geographical factors such as orientation [17]. Recent studies have also reported that bacterial diversity on the grape epidermis is effected by geographic patterns [17,41]. However, the diversity and richness of the bacterial community in the winery environment in the three sub-regions were the highest in the YC production area and the lowest in the YN sub-region, and the diversity and richness of the bacterial community in the winery environment were related to the use of commercial starter cultures and excessive manual intervention, so they were inconsistent with the diversity and richness of the bacterial community in the grape epidermis [19,42].

In this study, we found that the main bacterial phyla in the ripening stage of the grape epidermis in the three sub-regions of Helan Mountain were Proteobacteria, Firmicutes, Bacteroidetes and Actinomycetes. Yu-jie Wei et al. investigated the bacterial diversity on the surface of grapes by Illumina MiSeq and found that the main bacterial phyla in the grape epidermis were Proteobacteria, Firmicutes, Bacteroidetes, and Actinomycetes, which were consistent with the results of this study [14]. Proteobacteria, firmicutes, bacteroidetes, and actinomycetes have also been found to be dominant bacteria in soil, grape berries, and leaves [10,43,44,45,46,47], suggesting that these bacteria may originate in the natural environment and are somehow transferred to the grape skin. How bacteria from the natural environment are transferred to the grape skin and what impact they have on the wine needs to be further investigated.

The genera with a relatively high abundance of bacteria in the grape skin were *Massilia*, *Achromobacter*, *Hymenobacter*, *Pseudomonas*, *Sphingomonas*, *Enterobacter*, and *Pantoea*. Martins G and Fernandes P et al. found *Pantoea* and *Pseudomonas* to be the dominant bacterial genera on the skin of wine grapes [44,48]. Guilherme Martins et al. found that the most abundant genera on grapes, grape leaves, grape skins, and grape soils were *Pseudomonas* and *Sphingomonas* in 2013 [44]. This study also found large amounts of *Pseudomonas* and *Pantoea* on the skin of Cabernet Sauvignon grapes. These results suggest that these bacteria are widely distributed on the grape skin. However, due to geographical factors, grape varieties, and other reasons, the bacterial communities distributed on the grape epidermis are not the same. For example, in this study, *Hymenobacter*, *Enterobacter*, and a large quantity of *Achromobacter*, which is a genus typically found on the surface of berries [43], were also observed. These bacteria colonize the grape epidermis, and they certainly play a vital role in the health, development, yield, and quality of the grapes, and ultimately in the quality and organoleptic properties of the wine. Ye, Xiaofang et al. found that there was a significant correlation between *Achromobacter* and a variety of esters and alcohols in wine [49]. Zhang Zhong et al. found that there was a significant positive correlation between *Pantoea* and linear fatty alcohols, aromatic aldehydes, and terpenes [5]. Therefore, further in-depth research is needed on the interaction between grapes and bacteria and the influence of bacteria on grape juice and wine composition.

Studied have found that it was not only the geographical location that influences the microbial community on the grape skin, but also the climatic conditions (e.g., temperature, rainfall, wind, etc.) and vineyard management practices [3,8,9]. In this study, we investigated the correlation between the 10 most abundant bacterial genera and meteorological factors (temperature, humidity, wind speed, rainfall, and sunshine) in the three sub-regions, and found that there was a close interaction between bacteria and meteorological factors. In addition, vineyard management often uses pesticides to prevent grape pests and diseases, and studies have shown that pesticides could affect the grape epidermal microbial community [9,50], such as Cátia Pinto et al., which found that pesticides could lead to lower relative abundance values of grape epidermal bacteria (Enterobacteriaceae, Pseudomonas, Trichomonas or Xanthomonas family, etc.) [51]. Verginer et al. also found that the use of pesticides affected the bacterial community of the grape epidermis [52]. The authors have not yet studied the effects of pesticides on vineyard microorganisms, and in the future, we need to explore the changes in microorganisms caused by the use of pesticides in different production areas in the Eastern Foothills of Helan Mountain in Ningxia.

In the analysis of the bacterial composition and distribution of the environment of representative wineries in the three sub-regions of Helan Mountain, it was found that the bacterial genera *Achromobacter*, *Massilia*, *Sphingomonas*, *Acinetobacter*, and *Hymenobacter* were mainly enriched in the fermentation tank mouth, destemming crusher, air bag press, and conveyor belt of the winery, which were in close contact with grape juice and grapes. *Achromobacter*, *Massilia*, and *Sphingomonas* were also the dominant bacterial genera on the grape epidermis, among which *Achromobacter* was the most common and relatively abundant genus in the bacterial community of the grape epidermis in the three sub-regions, so the dominant bacteria in the grape fermentation process may mainly come from the grape epidermis. A further Venn analysis found that a large number of bacterial genera on the grape epidermis were present in the winery environment, and only a few dozen genera were exclusive to the grape epidermis, which indicates that grapes will bring a large number of bacteria into the winery and may be present in the fermentation process, and some studies have found that *Massilia* and *Sphingomonas* are also the dominant bacterial genera in the wine alcoholic fermentation process [5,19,20]. This indicates that the bacterial community of the grape epidermis may follow the grapes into the fermentation process and remain in the winemaking equipment and environment. Further experiments are needed to explore whether bacteria can exist in the fermentation process and how they can play a role in affecting the quality of grapes. Correlation analysis of the bacterial community found in grape epidermis and the bacterial community of the winery environment showed that there was no significant correlation between them, indicating that although the bacteria found in the grape epidermis could be brought into the winery, their abundance levels may change. Therefore, understanding the bacterial composition of the grape epidermis and the winery environment is conducive to ensuring grape health and promoting the transformation of grapes into wine, providing an important reference for exploring the effect of bacteria on the wine fermentation process and also helping to build a correlation between bacteria and wine quality.

## 5. Conclusions

In this study, about 93% of the bacterial genera comprising the grape epidermal bacteria in the three sub-regions of the Eastern Foothills of Helan Mountain in Ningxia were present in the winery environment, including *Achromobacter*, *Massilia*, *Sphingomonas*, and other dominant genera. However, in this study, the grape epidermis and the winery environment were not screened for bacteria that could be cultured, nor was wine fermentation carried out to explore the effect of bacteria on wine quality. In the future, we will study the relationship between the functional bacteria found in the grape skin and the flavor of wine.

## Data Availability

The original contributions presented in the study are included in the article/supplementary material, further inquiries can be directed to the corresponding author.

## Supplementary Materials

The following supporting information can be downloaded at: https://www.mdpi.com/article/10.3390/foods13020252/s1, Figure S1: The geographical location of vineyards in the three sub-regions; Figure S2. Correlation analysis of bacterial community diversity between grape skin and winery environment. Figure S3. Correlation analysis between the top 10 bacterial genera with relative abundance in grape skin and the top 10 bacterial genera with relative abundance in the winery environment. Table S1: Weather conditions of sub-region: Table S2: Bacterial diversity in the epidermis of grapes in different sub-regions; Table S3: Bacterial diversity in the winery environment in different sub-regions.
