# Supplementary material for: Diversity of ‘Cabernet Sauvignon’ Grape Epidermis and Environmental Bacteria in Wineries from Different Sub-Regions of the Eastern Foothills of Helan Mountain, Ningxia
Source: Foods. 2024 Jan 12;13(2):252. doi: 10.3390/foods13020252 (PMC10815095; doi:10.3390/foods13020252)
Supplement: Supplementary file 1 [file foods-13-00252-s001.zip › Drawings attached to the article.pdf]

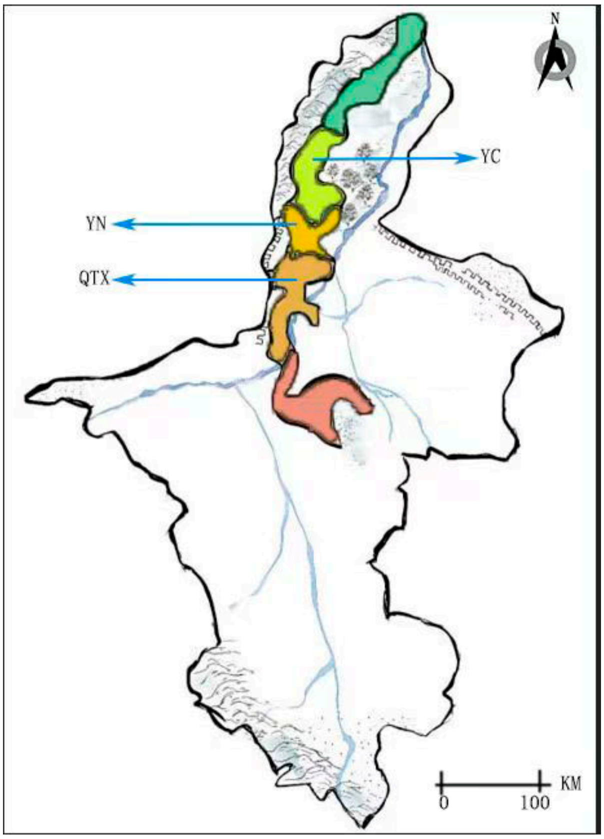

Figure S1. The geographical location of vineyards in the three sub-regions : YC, Yinchuan sub-region; YN, Yongning sub-region; QTX, Qingtongxia sub-region.

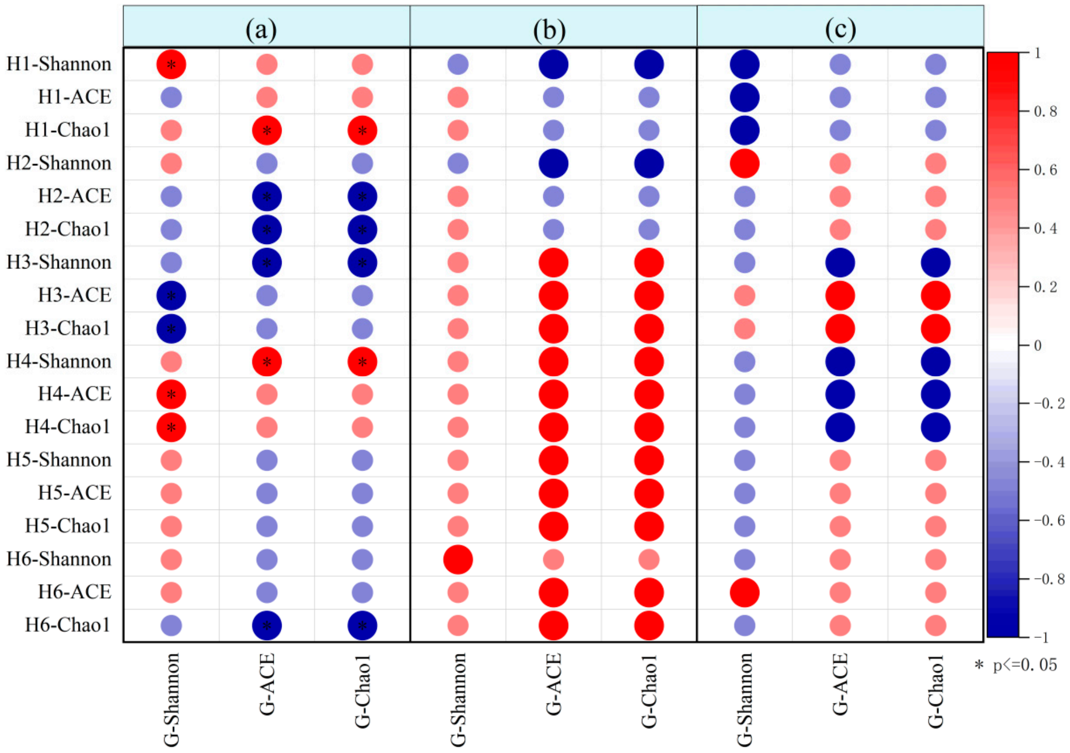

Figure S2. Correlation analysis of bacterial community diversity between grape skin and winery environment. A red circle with an asterisk indicates a positive correlation (Spearman rank test, *p* < 0.05), while a blue circle with an asterisk indicates a negative correlation (Spearman rank test, *p* < 0.05). X-axis, represents the bacterial diversity of the grape epidermis; Y-axis, represents the bacterial diversity of the winery environment.

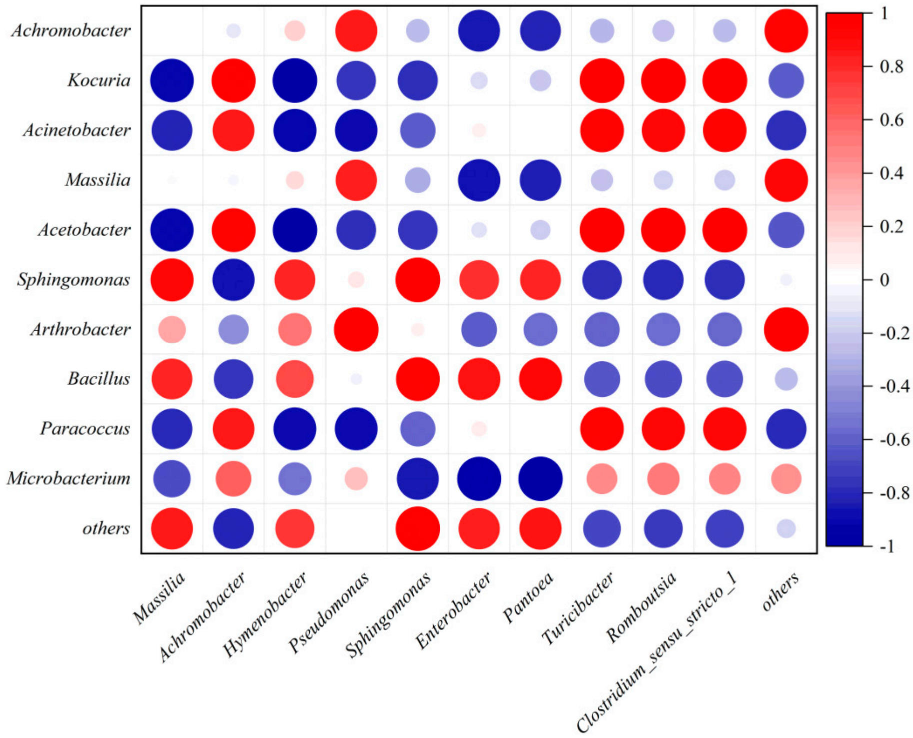

Figure S3. Correlation analysis between the top 10 bacterial genera with relative abundance of grape skin and the top 10 bacterial genera with relative abundance in winery environment. A red circle indicates a positive correlation, and a blue circle indicates a negative correlation. X-axis, represents the genus of grape epidermal bacteria; Y-axis, represents the genus of winery environmental bacteria.
